# Abdominal angiostrongyliasis in the Americas: fifty years since the discovery of a new metastrongylid species, *Angiostrongylus costaricensis*

**DOI:** 10.1186/s13071-021-04875-3

**Published:** 2021-07-22

**Authors:** Alicia Rojas, Arnaldo Maldonado-Junior, Javier Mora, Alessandra Morassutti, Rubens Rodriguez, Alberto Solano-Barquero, Anamariela Tijerino, Marianela Vargas, Carlos Graeff-Teixeira

**Affiliations:** 1 Laboratory of Helminthology, Centro de Investigación en Enfermedades Tropicales, University of Costa Rica, San José, Costa Rica; 2 Laboratório de Biologia e Parasitologia de Mamíferos Silvestres Reservatórios, Fundação Oswaldo Cruz – FIOCRUZ, Rio de Janeiro, RJ Brazil; 3 Instituto de Patologia e Biologia Molecular de Passo Fundo, School of Medicine, IMED Passo Fundo, Rio Grande do Sul, Brazil; 4 National Reference Center of Parasitology, Instituto Costarricense de Investigación y Enseñanza en Nutrición y Salud, Cartago, Costa Rica; 5 Nucleo de Doenças Infecciosas, Centro de Ciências da Saúde, Universidade Federal do Espírito Santo, Vitória, Brazil

**Keywords:** *Angiostrongylus costaricensis*, Abdominal angiostrongyliasis, Eosinophilic enteritis, Zoonosis, Helminthiasis

## Abstract

*Angiostrongylus costaricensis* is a zoonotic parasitic nematode described for the first time in 1971 by Pedro Morera and Rodolfo Céspedes in Costa Rica. This parasite causes an infection known as abdominal angiostrongyliasis, affecting mainly school-aged children and young adults. Infection with *A. costaricensis* has been associated with a myriad of rodent and mollusk species in the Americas and the Caribbean, as its natural hosts and reservoirs. In this commemorative review, we highlight the extensive research collected through a 50-year journey, which includes ecological, pathological, and molecular studies on *A. costaricensis* and its implicated disease. We also identify major knowledge gaps in its evolutionary history, the ecological role of imported and invasive mollusk species, and immune response. We propose that the advent of -omics analyses will allow us to gather novel information regarding *A. costaricensis* biology and infection dynamics, as well as to promote the design of much-needed sensitive and specific diagnostic tools. 
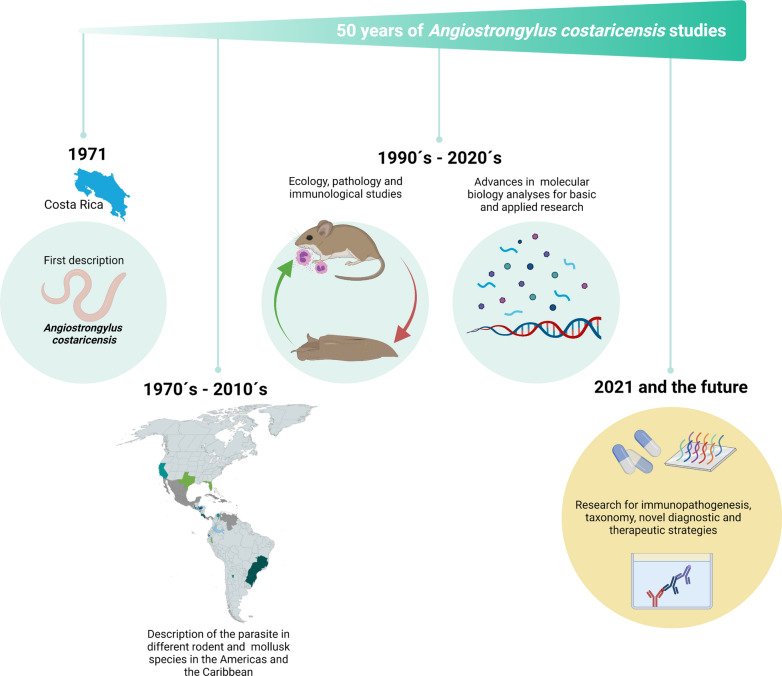

## Background

*Angiostrongylus costaricensis* is a zoonotic parasitic nematode that causes eosinophilic enteritis in humans, known as abdominal angiostrongyliasis (AA). Since its description in 1971 in Costa Rica, it has been reported from the southern regions of the United States to most Latin American countries and several Caribbean islands. The interest and concern that has promoted the study of *A. costaricensis* derive from the severe pathology that AA represents in humans, especially to school-age children and young adults, and the intricate life cycle that involves mollusks and rodents as intermediate and definitive hosts, respectively.

### History of *Angiostrongylus costaricensis*

The first case of AA in humans was described in 1952 in a seven-year-old child from Costa Rica suffering from severe abdominal pain with thickened intestinal serosa [1, 2]. Even though the etiological agent could not be identified at that time, the child was treated by surgical ileocecal resection and recovered completely. Over the next 16 years, 63 similar cases were received by the reference pathology laboratory from Costa Rica (Servicios de Anatomía Patológica, Hospital San Juan de Dios, and Caja Costarricense de Seguro Social) [1, 3], mainly derived from school-age children from all regions of the country. Most cases were characterized by severe abdominal pain in the right iliac fossa with fever and anorexia [3]. Analyses of the intra-abdominal masses recovered from these patients showed thickening in the appendix, hardening of an edematous intestinal wall, and yellowish granulomatous infiltration that led to partial to complete obstruction and necrosis. Complete blood counts were characterized by leukocytosis and eosinophilia that ranged from 11 to 81% of total leukocytes. In these cases, a detailed morphological analysis based on histopathology sections with the presence of male and female adult nematodes led to a preliminary identification of metastrongylid worms [2].

**Fig. 1:**
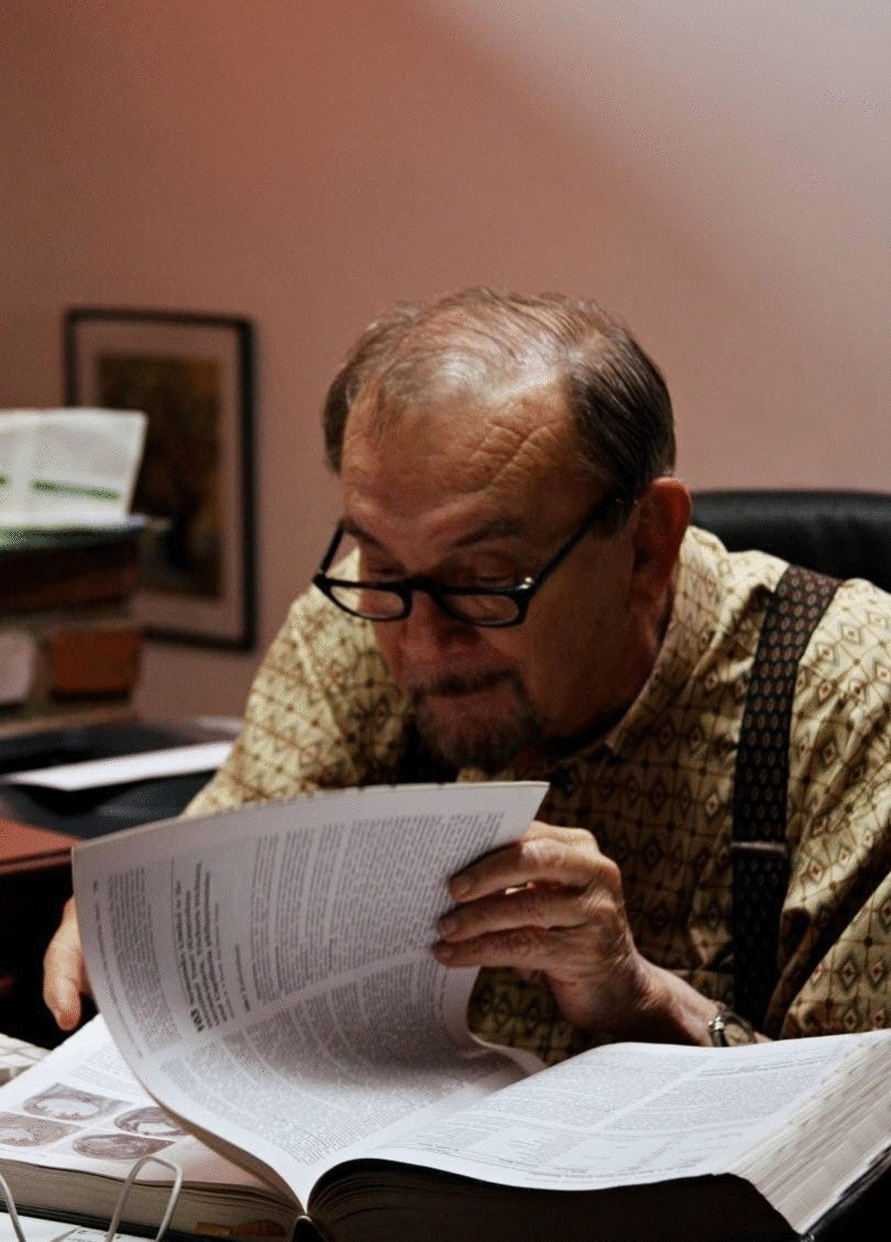
Pedro Morera, Emeritus Professor at the University of Costa Rica and Past President of the World Federation of Parasitologists. Together with Marta Conejo-Víquez and Arnoldo Castro-Araya, were pioneers in the study of the *Angiostrongylus costaricensis* life cycle, pathology, and ecology. Photo credit: Marcia Krauze Diehl. This photo is licensed under the Creative Commons CC BY license

Pedro Morera (Fig. 1) and Rodolfo Céspedes, two scientists from the University of Costa Rica and the San Juan de Dios Hospital, Costa Rica, respectively, finally characterized the nematode specimens obtained from clinical cases. In their report published in 1971, they described a new species in the genus *Angiostrongylus*, and proposed the name *A. costaricensis*. This characterization was based on several morphological differences from other members of the genus, including the size of the spicules and the presence of a terminal spine in females and a gubernaculum in males [4]. In that study, the authors highlighted that humans were not the natural hosts of the parasite, since larvae were not excreted in feces. *Angiostrongylus costaricensis* was compared to *Angiostrongylus cantonensis*, the other species infecting humans, and they hypothesized that the novel *A. costaricensis* was potentially better adapted to humans than the congeneric species, since adults were found within arterioles and larvated eggs were detected across tissue layers. Contrarily, during the eosinophilic encephalitis caused by *A. cantonensis*, third-stage larvae (L3) do not develop further and remain inside brain arterioles [5]. The following year, the first AA case outside Costa Rica was described in Honduras by Edgardo Sierra and Pedro Morera [6].

Dr. Pedro Morera concentrated all his efforts on elucidating the natural intermediate and definitive hosts of *A. costaricensis* in parallel with the description of human AA cases [7, 8]. In 1970, the cotton rat (*Sigmodon hispidus*) and the black rat (*Rattus rattus*) were found with *A. costaricensis* worms in their mesenteric arteries. However, the strong tissular reaction described in human cases was not observed in the animals. Instead, larvated eggs and L1 larvae were observed in the rat’s intestinal mucosa and L1 were shed in their feces [8]. In addition, the terrestrial slug *Sarasinula plebeia* (syn. *Vaginulus plebeius*) was identified as the intermediate host of the parasite after the recovery of L3 larvae from tissue digests [7]. These novel findings directed the reconstruction of *A. costaricensis*’s life cycle by performing experimental infections of laboratory-raised *S. hispidus* with L3 obtained from naturally infected *S. plebeia*. The careful analysis of the development of L1, L2, and L3 stages in slugs and the maturation of L3 into adults in rats led to a more detailed redescription of *A. costaricensis* in 1973 [9]. Fifty years after its original description, *A. costaricensis* has been reported in a myriad of rodent and slug species in more than ten countries in the Americas and still compromises the health of hundreds of individuals.

### Distribution and epidemiology

**Fig. 2:**
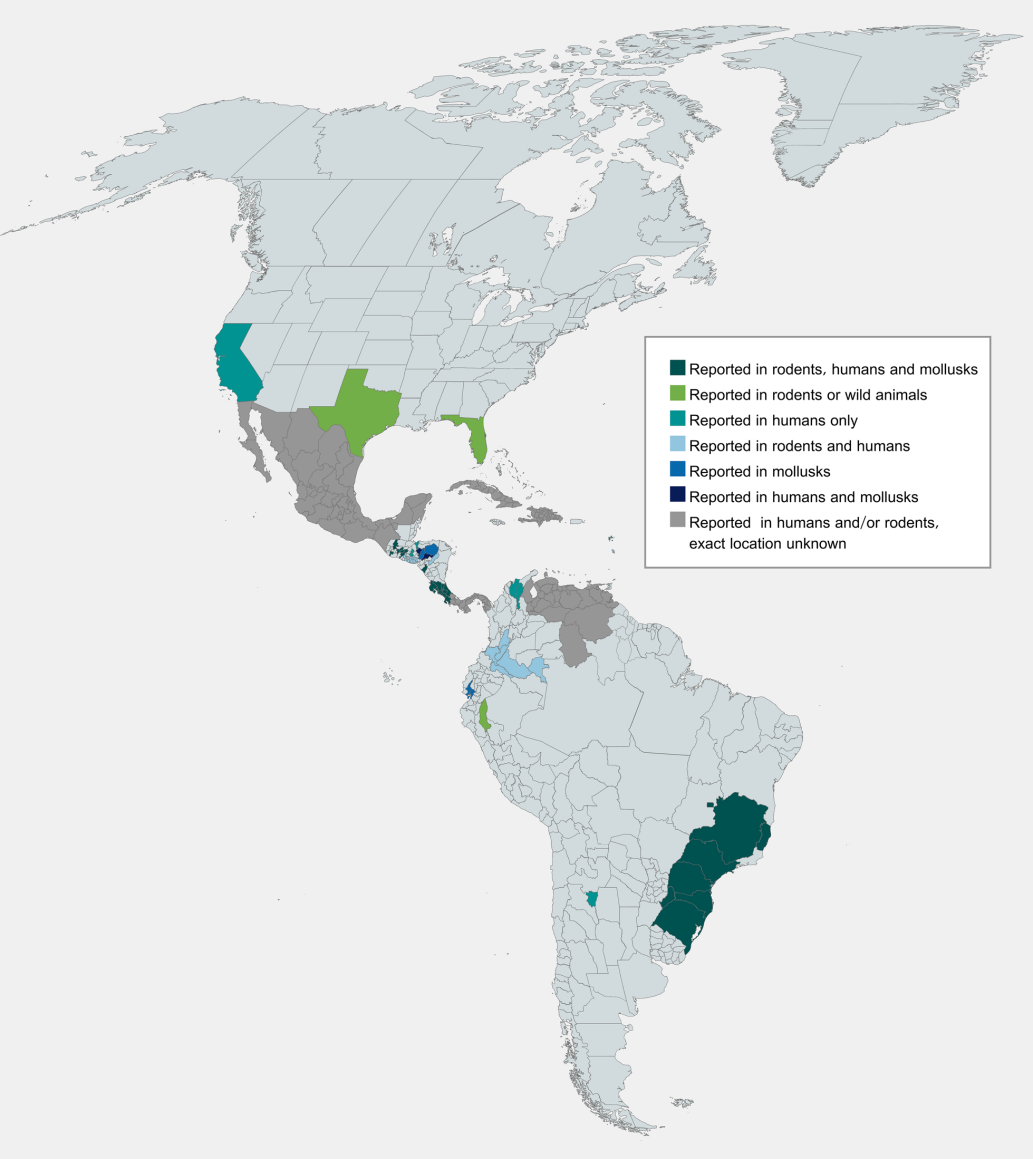
Geographical distribution of *Angiostrongylus costaricensis* in the Americas. This parasite has been detected in North and Central America in the USA (Los Angeles, Miami, Texas) [11, 53], Mexico (not specified in which location) [13], Guatemala (Chiquimula, Ciudad de Guatemala, El Progreso, Guastatoya, Jalapa, Jutiapa, Suchitepequez) [41], Honduras (Comayagua, Cortés, El Paraíso, Francisco Morazán, Olancho) [45], El Salvador (not specified), Nicaragua (León) [17, 62], Costa Rica (Alajuela, Cartago, Guanacaste, Heredia, Limón, Puntarenas, San José) [37], and Panama (not specified) [18]. In South America, *A. costaricensis* has been reported in Colombia (Caquetá, Huila, Putumayo, Tolima, Valle, Vaupés) [19], Venezuela (not specified) [20], Ecuador (Guayaquil) [22], Perú (Iquitos) [21], Brazil (Rio Grande do Sul, Santa Catarina, Parana, São Paulo, Brasília, Minas Gerais, Espírito Santo) [68, 86], and Argentina (Misiones, Tucumán) [24]. *Angiostrongylus costaricensis* has been found in several geographical locations in the Caribbean, including Cuba [25], Guadeloupe [29], Haiti [28], Martinique [27], and the Dominican Republic [26]. Finally, rare cases have been detected in Spain [35] and Zaire [32]. This figure was created using mapchart.net

*Angiostrongylus costaricensis* has been reported in 24 geographic sites from the Americas and the Caribbean [10] (Fig. 2), either in their rodent definitive hosts, causing AA in humans, or in mollusk intermediate hosts. The parasite has been detected in the USA [11, 12], Mexico [13], Guatemala [14], Honduras [6, 15], El Salvador [16], Nicaragua [17], Costa Rica [9], Panama [18], Colombia [19], Venezuela [20], Peru [21], Ecuador [22], Brazil [23], Argentina [24], Cuba [25], the Dominican Republic [26], Martinique [27], Haiti [28], and Guadeloupe [29]. These geographical locations meet abiotic criteria for the optimal development of *A. costaricensis* in their definitive and intermediate hosts [30], such as warm temperatures, abundant or moderate rain [31], and diverse vegetation. In addition, rare infections in Zaire [32], and imported cases in two different patients from the USA visiting El Salvador [33, 34] and two independent Spanish individuals with a history of travel to Nicaragua [35, 36] have also been described.

This parasite is considered endemic in Costa Rica, since hundreds of human individuals tested positive between 2012 and 2020 using a latex agglutination test [37]. Furthermore, this Central American country has the highest concentration of reported cases in the world, while only a few cases have been detected in the other locations [38], suggesting the infection is underdiagnosed in other regions, especially due to lack of awareness of angiostrongyliasis as a cause for eosinophilic gastroenteritis. Interestingly, a longitudinal study that followed individuals between 1995 and 1999 in southern Brazil found a yearly prevalence as high as 28.2% [39]. Altogether, these studies indicate the high risk of infection in humans as accidental hosts and the great burden it causes to vulnerable populations.

Cases of AA are usually more abundant in males than in females. A higher incidence of AA has been observed in males from Costa Rica [37], Guatemala [40], Nicaragua [17], Martinique, and other islands of the Greater Antilles [27], but no gender tendency has been detected in a more recent study from Guatemala [41] or other geographical locations. Furthermore, in the 1980s, individuals older than 18 years were the most heavily affected with infection in Costa Rica. In contrast, from 2000 to 2020, the highest incidence was found in children younger than 15 years in Costa Rica [37, 42], Colombia [43, 44], and Martinique [27]. This shift in age might be explained by the underdiagnosis in children in the first study, or increased awareness among adult populations about the parasite. Nevertheless, an outbreak of 22 AA cases in Guatemala between 1994 and 1995 identified a median age of 37 years [40], and a subsequent study in this same country showed that infection was more common in individuals older than 16 years [41]. In addition, AA cases have not been associated with low socioeconomic status or with low nutritional condition [3]. These studies demonstrate a general pattern of vulnerable populations that might differ among geographical regions.

A higher number of cases are reported during the rainy season (June to November) in Costa Rica [1, 3], Honduras [45], Colombia [43], and Martinique [27], when intermediate host populations are present in greater numbers. However, in Guatemala, most cases occur in January and February, except in the 1995 outbreak, where infections were detected mainly in May and were associated with mint consumption [41].

### Life cycle

#### Definitive hosts

**Fig. 3:**
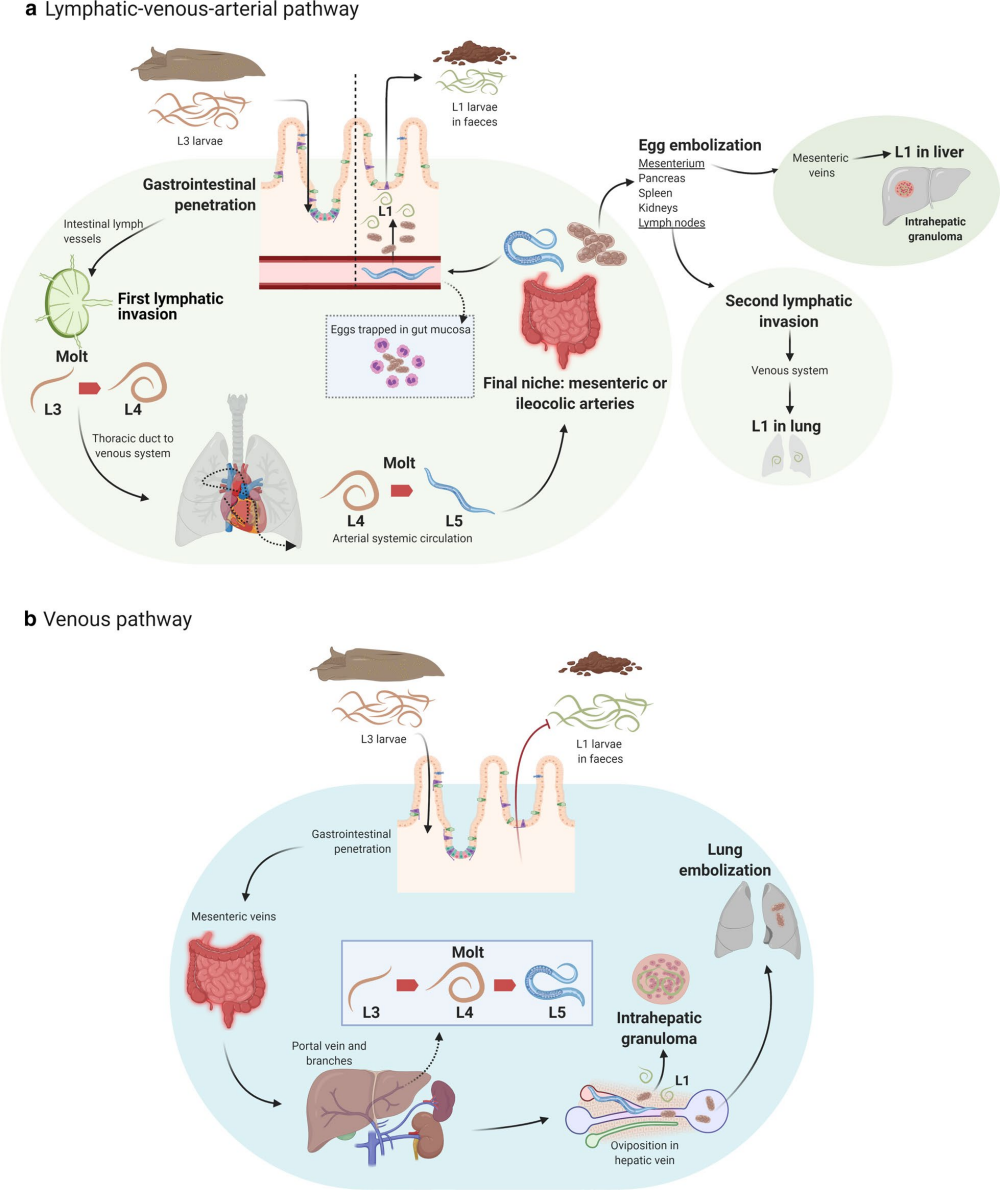
Migratory courses of *Angiostrongylus costaricensis* L3 larvae in experimental infection of cotton rats *Sigmodon hispidus*. **a** The lymphatic–venous–arterial pathway that leads to the development of adult worms in mesenteric or ileocolic arteries and excretion of L1 larvae in the rat’s feces. **b** Depicts the venous course, which is less common, and adult stages are found in hepatic veins. In this pathway, L1 larvae are not shed in feces. This figure was created using BioRender.com

*Angiostrongylus costaricensis* has an indirect life cycle involving rodents as definitive hosts and gastropods as intermediate hosts. Definitive hosts become infected by ingesting L3 larvae present in fibromuscular tissues from infected gastropods or having contact with their slime-containing L3 larvae [46]. Then, L3 larvae are released in the stomach, penetrate the gastrointestinal mucosa, and follow two migratory courses during their development into male and female adults. The main pathway, known as the lymphatic–venous–arterial course, involves the passage and concomitant molting of the worms in the lymphatic system and arterial systemic circulation until reaching their final niche in the mesenteric or ileocolic arteries (Fig. 3). From there, males and females copulate and produce hundreds of eggs that are transferred and hatched in the gut mucosa as L1 larvae. The latter are released in the intestinal lumen by active movement or expelled with necrotic detritus from day 27 after infection [47]. Then, L1 are shed in the environment in the rodent’s feces, which can remain viable for infection of mollusks for up to 10 days [48]. The second migratory route, known as the venous pathway, is less common, and L3 larvae reach the mesenteric veins after gastrointestinal penetration. After this, they reach the portal vein and its branches in the liver until maturating to adult stages. Released eggs induce intrahepatic granulomas or are embolized to the lungs [47]. This pathway has relevant ecological implications since L1 larvae are not released in the rodent’s feces, thus impairing the progression of the *A. costaricensis* life cycle.

*Sigmodon hispidus* has been implicated as the natural definitive host of the parasite. However, other rodent species such as *Rattus norvegicus* [8], *R. rattus* [16], *Peromyscus* spp. [16], *Lyomis adspersus* [16], *Oligoryzomys fulvescens* [16], *Oligoryzomys nigripes* [49], *Oryzomys ratticeps* [50], *Akodon montensis* [51], *Zygodontomys brevicauda* [16], and *Melanomys caliginosus* [19] are also competent definitive hosts of the parasite in different geographical locations and perpetuate the circulation of *A. costaricensis* in the environment [16] (Table 1). Importantly, different rodent species have distinct susceptibility to *A. costaricensis* infection; for example, infected wild *Mus musculus* strains have demonstrated high morbidity and mortality [52] when compared to wild rodents such as *S. hispidus*, *O. nigripes*, or *O. ratticeps.* Therefore, policies must be issued towards controlling rodent populations in agricultural fields, where mollusk intermediate hosts are also abundant and maintain the life cycle of this parasite.

Other wild animals have been suggested as potential hosts with *A. costaricensis* in captivity or in their wild habitats. For instance, captive raccoons (*Procyon* lotor), siamang monkeys (*Hylobates syndactylus*), Nancy Ma’s night monkeys (*Aotus nancymaae*), black-chested mustached tamarins (*Saguinus mystax*) [53], a black-tufted-eared marmoset (*Callithrix penicillata*) [21], and an opossum (*Didelphis virginiana*) [53] were found with adult parasites in their mesenteric arteries. However, these reports have been based on microscopic and histopathological identification of the parasites from lesions, and not from detailed morphometric and molecular analyses. Therefore, the true taxonomic status of some specimens is uncertain. Other vertebrate species, such as domestic dogs [54] and white-nosed coatis (*Nasua narica*) [55, 56] from Costa Rica, have been reported as potential reservoirs of the parasite based on the finding of *A. costaricensis*-like specimens in feces and mesenteric arteries that exhibited high sequence similarity to *A. costaricensis* (99–100%). However, *Angiostrongylus minascensis*, a newly described species found in *Nasua nasua* from Brazil, was 100% identical to the specimens obtained from Costa Rican coatis [57]. Altogether, these analyses suggest that wild hosts might harbor separate *Angiostrongylus* spp., and the diversity of the genus *Angiostrongylus* is likely to be greater than currently reported (Table 1).

**Table 1:** Vertebrate species reported as *Angiostrongylus costaricensis*-competent hosts based on morphological or molecular identification of the parasite

Vertebrate host	Order and family of host	Country	Diagnostic method	References
*Sigmodon hispidus*	Rodentia, Cricetidae	Costa Rica, Honduras, El Salvador, Panama, Brazil, USA	Histopathological examination	[8, 16, 18]
*Rattus rattus*	Rodentia, Muridae	Costa Rica, Panama, Guadeloupe	Histopathological examination	[8, 18, 29]
*Rattus norvegicus*	Rodentia, Muridae	Guadeloupe	Histopathological examination	[29]
*Peromyscus* spp.	Rodentia, Cricetidae	Honduras	NI	[16]
*Lyomis adspersus*	Rodentia, Heteromyidae	Panama	Histopathological examination	[18]
*Oligoryzomys fulvescens*	Rodentia, Cricetidae	Panama, Brazil	Histopathological examination	[16, 18]
*Oligoryzomys nigripes* (syn. *Sooretamys angouya*)	Rodentia, Cricetidae	Brazil	Histopathological examination	[139]
*Oryzomys ratticeps*	Rodentia, Cricetidae	Brazil	Histopathological examination	[139]
*Akodon montensis*	Rodentia, Cricetidae	Argentina	Histopathological examination	[51]
*Zygodontomys brevicauda*	Rodentia, Cricetidae	Panama	NI	[10]
*Melanomys caliginosus*	Rodentia, Cricetidae	Colombia	Morphological identification of L1 in rodent feces	[19]
*Procyon lotor*^a^	Carnivora, Procyonidae	USA	Histopathological examination	[53]
*Hylobates syndactylus*^a^	Primates, Hylobatidae	USA	Histopathological examination	[53]
*Aotus nancymaae*^a^	Primates, Aotidae	USA	Histopathological examination	[53]
*Saguinus mystax*^a^	Primates, Callitrichidae	Peru	Histopathological examination	[21]
*Callithrix penicillata*^a^	Primates, Callitrichidae	Brazil	Histopathological examination	[53]
*Didelphis virginiana*^a^	Didelphimorphia, Didelphidae	USA	Morphological identification	[53]
*Nasua narica*^b^	Carnivora, Procyonidae	Costa Rica	Histopathological examination and molecular analysis	[55]
*Canis lupus familiaris*	Carnivora, Canidae	Costa Rica	Histopathological examination and molecular analysis	[54]

NI, not indicated in the source

^a^These animals were kept in captivity

^b^The specimens collected from this work were later identified as *Angiostrongylus minascensis*

Humans are considered accidental or dead-end hosts of *A. costaricensis* because eggs or L1s are not released in feces. Instead, a strong inflammatory response is induced in the intestinal serosa and halters the mobilization of the eggs to the lumen [4]. Humans may become infected when ingesting the mollusks hidden in vegetables or less likely from L3s released in the mollusk’s slime [16]. Infective larvae are suggested to follow the lymphatic–venous–arterial pathway, although cases of hepatic granulomas as a result of a venous pathway migration have also been described [58]. Additionally, the incubation period of AA in humans is currently unknown, but it has been estimated to range from three weeks to several months [59].

### Intermediate hosts

#### Diversity of intermediate hosts

*Angiostrongylus costaricensis* uses terrestrial gastropod mollusks of eight different taxonomic families as intermediate hosts [60] (Table 2), indicating a low host specificity [61]. At least 18 gastropod species have been detected with L3 larvae in natural and experimental settings including *S. plebeia* in Costa Rica [7], Nicaragua [62] and Ecuador [22], *Phyllocaulis variegatus* [63], *Limax* spp. [61] and *Bradybaena similaris* [61] in Brazil. Most of these reports are derived from the morphological identification of *A. costaricensis-*like L3 larvae in infected mollusks with subsequent infection of rat laboratory models. However, those reports did not recover adult worms in experimentally infected animals and might have misidentified the obtained L3 larvae from the closely related species *A. cantonensis* or *A. minascensis*, since 11 out of 18 known *A. costaricensis*-intermediate hosts have been reported also as competent hosts for *A. cantonensis.* Moreover, *Biomphalaria glabrata* [64], *Biomphalaria straminea* [65], *Biomphalaria tenagophila* [65] and *Sarasinula linguaeformis* [66] have been found naturally infected with *A. costaricensis* and *A. cantonensis.*

**Table 2:** Mollusk species reported as *Angiostrongylus costaricensis* competent hosts based on morphological identification of the parasite

Host species	Family	Host type	Geographical location	Percentage of maximum prevalence reported in naturally infected samples (total sample)	Maximum parasitic L3 load reported in naturally infected samples	Type of infection reported in mollusks	References
*Belocaulus angustipes*^a^	Veronicellidae	Land slug	Brazil	33.3 (24)	13	Natural	[68]
*Biomphalaria glabrata*^a^	Planorbidae	Aquatic snail	Brazil	NNI	NNI	Experimental	[140]
*Biomphalaria straminea*^a^	Planorbidae	Aquatic snail	Brazil	NNI	NNI	Experimental	[140]
*Biomphalaria tenagophila*^a^	Planorbidae	Aquatic snail	Brazil	NNI	NNI	Experimental	[141]
*Bradybaena similaris*^b^	Bradybaenidae	Land snail	Brazil	93.4 (91)	98	Natural	[61]
*Cornu aspersum*^b^	Helicidae	Land snail	Brazil	4.7 (63)	ID	Natural	[68]
*Deroceras laeve*^b^	Limacidae	Land slug	Brazil	0.1 (9)	1	Natural	[67]
*Limax flavus*^b^	Limacidae	Land slug	Brazil	17 (12)	ID	Natural	[61]
*Limax maximus*^b^	Limacidae	Land slug	Brazil	28 (143)	ID	Natural	[61]
*Megalobulimus abbreviatus*^a^	Megalobulimidae	Land snail	Brazil	ID	ID	Natural	[61]
*Meghimatium pictum*^b^	Philomycidae	Land slug	Brazil	4.5 (245)	8	Natural	[69]
*Omalonyx* spp. ^a^	Succineidae	Semiaquatic slug	Brazil	NNI	NNI	Experimental	[72]
*Phyllocaulis boraceiensis*^a^	Veronicellidae	Land slug	Brazil	NNI	NNI	Experimental	[46]
*Phyllocaulis soleiformis*^a^	Veronicellidae	Land slug	Brazil	16.6 (6)	1	Natural	[68]
*Phyllocaulis variegatus*^a^	Veronicellidae	Land slug	Brazil	28 (100)	75	Natural	[63]
*Sarasinula linguaeformis*^a^	Veronicellidae	Land slug	Brazil	86 (50)	7720	Natural	[142]
*Sarasinula plebeia*^a^	Veronicellidae	Land slug	Costa Rica, Nicaragua, Honduras, Ecuador	69.3 (856)	4600	Natural	[9, 62, 70]
*Veronicella cubensis* (syn. *V. occidentalis*)^a^	Veronicellidae	Land slug	Colombia	NNI	NNI	Experimental	[19]

NNI, no natural infection reported

ID, insufficient data

^a^Native species

^b^Introduced species

The prevalence and load of *A. costaricensis* larvae in intermediate hosts vary across studies. However, veronicellids are considered highly permissive in the development of this parasite, as high numbers of L3 larvae have been recovered in different studies [9, 61, 62, 67–69] (Table 2). An exception to this is *B. similaris* of the family Bradybaenidae, which has presented a high prevalence of *A. costaricensis* infection [61]. The high diversity of susceptible intermediate hosts highlights the need to expand our understanding of the potential involvement of other species in the transmission of the parasite to humans in other settings.

Other physiological factors such as slug species and age of the host seem to influence *A. costaricensis* development. It has been demonstrated that this parasite is more prevalent in older and heavier slugs [70]. In addition, susceptibility and mortality due to *A. costaricensis* infection in mollusks vary between different species inoculated with L1 larvae, as shown in *Phyllocaulis* spp. [46]. *Phyllocaulis variegatus* was found to harbor more L3 larvae and had higher survival rates than *P. soleiformis*, suggesting a better coadaptation between the mollusk and the parasite [46].

### Studies on the infection of the mollusk and mollusk immune response

**Fig. 4:**
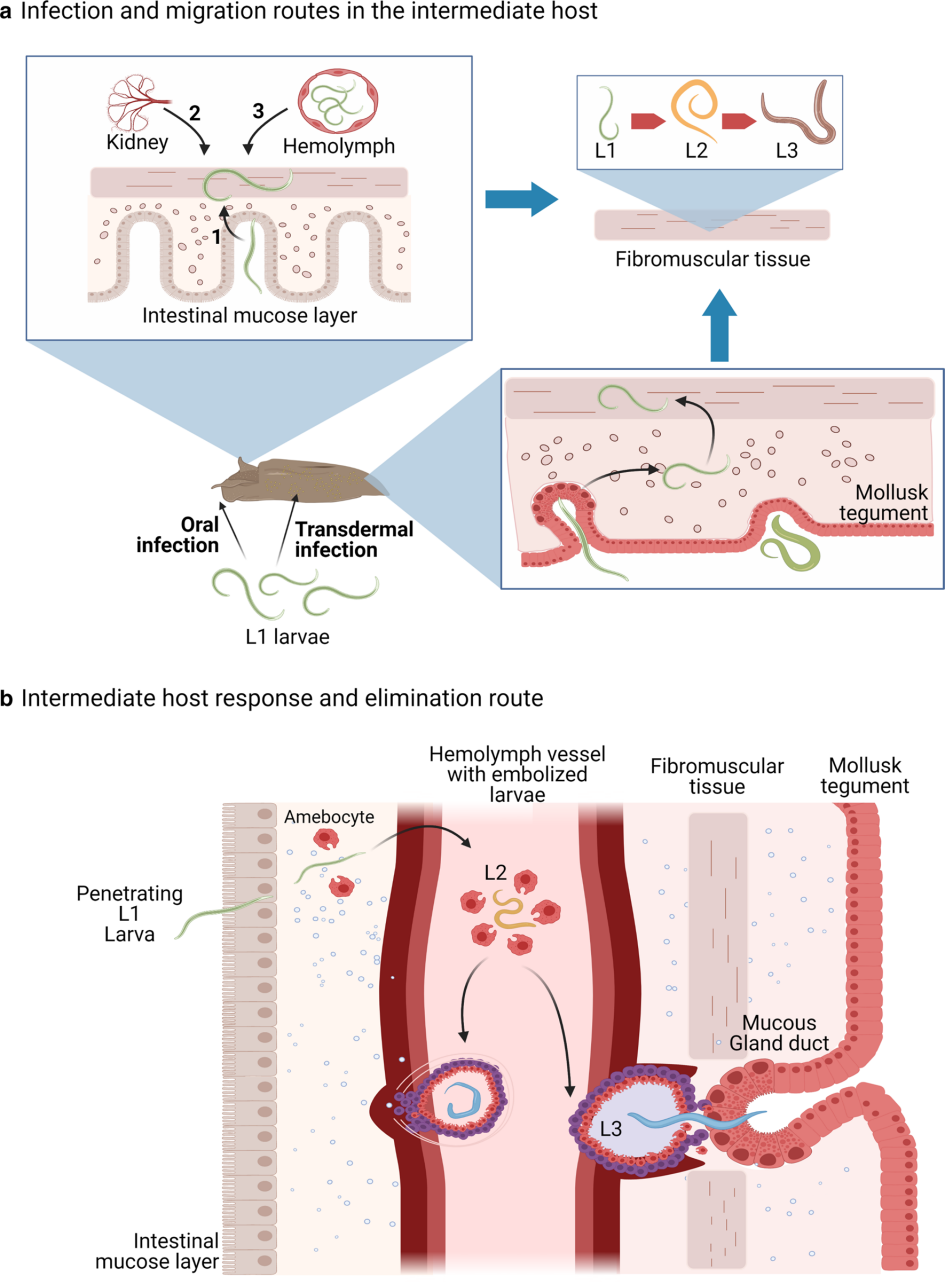
Proposed model of *Angiostrongylus costaricensis*–intermediate host interactions. These events are based on *Sarasinula linguaeformis* (syn. *S. marginata*) and *Omalonyx* spp. experimental infections [9, 72, 74–76]. **a** L1 larvae can infect mollusks using two main pathways: transdermal or oral infection. In the first, L1 larvae penetrate the mollusk’s tegument through the mucous gland pores and ducts. Then, larvae reach fibromuscular tissues by direct migration or embolization in hemolymph vessels to the kidney and other viscera. Molting to L2 stage occurs after 4 days of infection. During oral infection, L1 larvae reach fibromuscular tissues by penetrating the oral, intestinal, or rectal mucosa (1), through the kidneys (2), or through the hemolymph vessels (3). Only L1 larvae located in fibromuscular tissues molt until L3 stage. **b** After L1 larvae penetrate mollusk tissues and reach hemolymph vessels, either by oral or transdermal routes, poor perilarval granulomatous reactions with amebocytes are started after two to six hours post-infection. L1 to L2 transitioning is a strong cellular stimulus for amebocyte proliferation in the hemolymph. As the cellular response progresses, it is possible to observe different degrees of granulomas around the L2s, some of which become epithelioid cells. Some granulomas containing L2 larvae or in L2–L3 transition larvae will be located on the wall of vessels adjacent to different fibromuscular tissues, especially those anatomical regions juxtaposed with the acinus and ducts of the mucous glands of the tegument. In these cases, granulomatous reactions will degenerate hemolymph vessels and glandular tissues, giving the already mature and mobile L3 larvae access to glandular ducts in order to be shed in the mollusk’s mucous secretions. This figure was created using BioRender.com

Mollusks become infected with *A. costaricensis* by oral [9] and transtegumentary [71] routes in *S. plebeia* and *Sarasinula linguaeformis* (syn. *Sarasinula marginata*) (Fig. 4a), although direct inoculation of L1 larva into the body of the slug has also been demonstrated in *Phyllocaulis* spp. [46]. Thirty minutes after oral ingestion, L1 larvae become non-motile, disappear from the digestive tract with no preference in the specific site [72], and localize mainly at the foot and mantle of the slug, and rarely in the visceral mass. Four hours later, larvae reach the superficial layer of the mucous epithelium and even subepithelial connective and fibromuscular tissues [73]. L1 and L2 stages start accumulating internal granules, increase their size, and molt to L2 and L3 larvae after 4 and 11 days post-infection, respectively [9].

Cellular responses against *A. costaricensis* larval stages occur in the mollusk soon after infection [74, 75] (Fig. 4b). Amebocytes proliferate around larvae, and it has been suggested that this process is inversely correlated with the positioning of the parasites to acinar glands of the mollusk responsible for producing the mucus from the foot. This might indicate that the closer the larvae are located to the acinar glands, the less cellular response is produced against the parasites, and might indicate a possible larval modulation to amebocyte response to allow their release from the mollusk [70]. Subsequent experimental infections in the succineid semi-slug *Omalonyx* spp. have demonstrated that large numbers of hemocytes surrounding L1 larvae starts four hours after infection and weakens after the release of L3 stages [73, 75].

The main mechanisms of defense against *A. costaricensis* infection in mollusks are cellular granulomatous responses. Interestingly, this mechanism seems to be non-fatal to larvae in *S. linguaeformis* slugs and helps them to continue the life cycle of the worm [72, 74]. Over time, granuloma formation near gland ducts and the displacement of granulomas within the mollusk can help the larvae reach the circulatory system and then the fibromuscular layer by embolization of L3 stages [76] (Fig. 4b). Furthermore, shedding of non-motile L3 larvae can be facilitated with the granulomatous reaction that can disrupt nearby tissues or by the active release of aspartic proteases and metalloproteases by the worm [77].

L3 larvae are transmitted to definitive hosts mainly by the ingestion of their tissues [46, 71]. Alternative infection routes such as contamination of food with the slime have been suggested. However, L3 are also found in slime at a rate of 2 L3/g/day, while the number of L3 obtained from fibromuscular tissues reaches 162 L3/g of tissue [46]. These findings suggest that *A. costaricensis* transmission to definitive or human hosts by slime might constitute a rare event, and infection risk by this route needs to be assessed.

### Invasive and introduced mollusk species

Six out of the 18 slug species reported as intermediate hosts of *A. costaricensis* are introduced or invasive in the location where they were reported, and some of them are also hosts of *A. cantonensis*. The role that these introduced species have in spreading the parasite has not been well established for *A. costaricensis*, but the explosive spread of some mollusk species has raised concerns about this possibility [78].

Introduced and invasive mollusks that serve as intermediate hosts of *A. cantonensis* might represent new hosts for *A. costaricensis* and might expand the infection range to other geographical locations*.* The presence of *A. cantonensis* in Cuba, Puerto Rico, Colombia, and Brazil might have been facilitated by the introduction of infected *Rattus norvegicus.* However, its expansion also could have been favored by the presence of introduced mollusks known to be important intermediate hosts of *A. cantonensis*, such as terrestrial slugs *Limax maximus* or *Bradybaena similaris*, or the giant African snail *Achatina fulica* [79, 80]. The latter was introduced to the tropics and subtropics at the beginning of the nineteenth century and became an important pest in several crops. In Brazil, this species has spread to at least 23 out of the 26 Brazilian states [78, 80]. Nevertheless, the role of giant African snails as natural hosts of *A. costaricensis* has been debated. While some studies have found heavily infected *A. fulica* specimens [79], others have not recovered *A. costaricensis* larvae from them [81]. This can be explained by the high loads of L1 larvae required to infect *A. fulica* mollusks, and the fact that infection occurs only in a low percentage of the snails [82]. On the other hand, *B. similaris* is an invasive land slug native to Eastern Asia, a competent intermediate host of *A. cantonensis* [66], and was introduced to Colombia, Argentina, Paraguay, and Brazil [83]. This mollusk has spread in some regions of Brazil, where infection with *A. costaricensis* has been reported [68]. Many of the introduced mollusks are agricultural and garden pests; therefore, it is essential that their role in the dissemination and transmission of *A. costaricensis* be investigated.

The lack of specificity of *A. costaricensis* infection in mollusk hosts highlights the importance of investigating other mollusks and non-mollusk species that may act as intermediate or paratenic hosts. Importantly, *A. cantonensis* L3 larvae have been found in potential paratenic hosts such as centipedes from China and [84] monitor lizards *Varanus bengalensis* from Thailand [85] and are associated with eosinophilic meningitis in humans. Further research is needed on the potential paratenic hosts of *A. costaricensis* and their role in L3 larvae transmission to humans and in the development of AA.

### Molecular biology of the parasite

Proteins are key molecules in understanding metabolic functions, host–parasite interactions, antigen recognition, and possible sources for therapy targets. Many studies have been focused on the identification of *A. costaricensis* antigenic markers that could be used for diagnosis, but not intending to characterize the gene or protein sequences [86–89]. By using one-dimensional electrophoresis separation and Edman digestion, the low molecular weight proteins, glutathione S-transferase and ubiquitin, proved to be recognized by IgG and IgG1 of infected rodents [90]. With the improvements in protein separation methods such as two-dimensional electrophoresis (2D) combined with mass spectrometry (MS), Leon et al. [91] analyzed 49 selected spots from 2D gels. The obtained peptides allowed the identification of 20 proteins, among them galectins, heat shock proteins, myosin, aspartyl protease inhibitor, and annexin family protein [91]. At that time, this approach proved to be useful given that no genomic information for *A. costaricensis* was available in the commonly used NCBI non-redundant protein database. Later, approximately 1000 spots and differentiated protein profiles of male and female adult worms were identified by a 2D tandem MS technique [92], most of these involved in stress response and energy metabolism, as well as structural proteins. Also, this study identified 7.5% of spots exclusively found in females and 10.4% spots unique to male adult worms [92]. However, it was not possible to determine any biological role for those differences. One major limitation of this approach is the need for a great amount of a given molecule. Most proteins expressed in cells will not have detectable amounts in a 2D gel. Therefore, only the most abundant proteins will be identified.

Other studies have assessed possible functions and applications of the identified proteins by using protein activity assays [77, 93] and cloning parasite sequences into plasmids [94]. A synthetic peptide of the serine/threonine phosphatase 2A (PP2A), an enzyme involved in the embryogenesis and differentiation of *A. costaricensis*, was tested as a vaccine candidate in murine hosts [94]. This synthetic peptide conferred 60 to 100% protection against *A. costaricensis* challenge, with a significant increase in IFN-γ and IL-17 levels [94]. In addition, the proteolytic activity of this worm’s peptides was investigated and showed significant differences among the proteins derived from adult worms and L1 and L3 larvae [93]. These findings revealed important factors of the complex infection process and promoted different views of potential alternative methods of disease treatment and interruption of life-cycle transmission.

Molecular tools using homologous proteins from *A. cantonensis* have been useful for diagnosing AA [95] and for species differentiation [96, 97]. A previous study designed a conventional PCR targeting a 232-bp region of a 66-kDa native protein of *A. cantonensis* from sera of infected human patients diagnosed with AA [95]. This tool proved to be efficient for early detection during the acute stages of the disease when antibodies may not yet be detectable. However, only three samples were used, and validation remains to be established. This same target was also proposed for detecting *A. costaricensis* DNA in formalin-fixed paraffin-embedded (FFPE) tissues from infected patients, which could be useful given its high sensitivity and specificity, and may be helpful for those cases where no parasitic structures are found [98].

Molecular differentiation of *Angiostrongylus* spp. has been attempted using mitochondrial and ribosomal DNA (rDNA) loci. Assays were developed involving PCR amplification of the second internal transcribed spacer (ITS-2) and cytochrome oxidase I (*cox*1) followed by restriction fragment length polymorphism (RFLP) [96]. Distinct band patterns derived from several restriction enzymes were able to differentiate *A. costaricensis* from *A. cantonensis* and *A. vasorum* adult worms and larvae. This tool proved useful for rapid species identification when coinfection was suspected [96].

Different genetic markers have been used to analyze the phylogenetic relationships between *A. costaricensis* and other *Angiostrongylus* species*.* A study using the whole sequence of a 66-kDa protein proposed that *A. costaricensis* was the most distant taxon and the earliest divergent group when using *A. cantonensis* and *Angiostrongylus malaysiensis* sequences [99]. Additionally, *cox*1 sequences of *Angiostrongylus* spp. were analyzed and revealed a p-distance of 11.39% between Brazilian and Costa Rican *A. costaricensis* isolates, suggesting a possible cryptic differentiation in this parasite [97]. This high nucleotide difference was corroborated in the complete mitochondrial genomes of two isolates from Brazil and Costa Rica, which showed a p-distance of 16.2% using 12 protein-coding genes (PCG). Therefore, the taxonomic status of *A. costaricensis* should be further studied, in order to elucidate whether isolates from different geographical locations might represent cryptic or separate species.

The mitochondrial genome of *A*. *costaricensis* shed slight molecular light on this parasite [100, 101]*.* These studies revealed that the *A. costaricensis* mitochondrial genome encodes for 12 proteins, 22 transfer RNAs, and two ribosomal RNAs, and is considered the smallest characterized mitochondrial genome in the Chromadorea class. A difference in the size of the control region was also found between isolates of Brazil and Costa Rica, of 265 bp and 318 bp, respectively [100]. These data, together with the higher adenine/thymine (A-T%) content in control regions, rRNA genes, and most PCGs, suggest that *A. costaricensis* might constitute a species complex [100].

Only after the efforts of the 50 Helminth Genomes Project by the Wellcome Sanger Institute were the whole genomic sequences of *A. costaricensis* obtained, increasing the number of sequences deposited in GenBank enormously (BioProject: PRJEB494). Today, we may find 13,418 records of protein sequences and 6384 scaffolds of *A. costaricensis*. However, these data are far from elucidating the genome of *A. costaricensis*. First, it is necessary to assemble the genome which will derive data about the functional characteristics of this parasite, as has been analyzed for other nematodes [102, 103]. The investigation of the metabolic map and its comparison among susceptible definitive hosts will shed light on essential survival routes of the parasite. It may also reveal novel treatment strategies and pathogenesis, and increase our understanding of host and parasite interplay. Also, exploring the molecular sequences of transposons may help to understand the evolutionary history of *Angiostrongylus* and resolve the species complex suggested in previous studies [100]. The characterization of protein sequences and their posttranslational modifications such as glycosylation will be crucial for assessing antigenicity, as also analyzed for *A. cantonensis* [104]. Finally, de novo sequencing of proteins and mRNA from different stages of the life cycle will enable mapping of the proteins expressed in a particular moment of the infection and will help to better elucidate the richness of the molecular biology of *A. costaricensis*.

### Clinical presentation

Most patients with AA complain of abdominal pain, either spontaneous or induced by palpation, especially at the right iliac fossa and the right flank. This clinical symptom is in accord with the usual localization of *A. costaricensis* adult worms in the ileocecal branches of the inferior mesenteric artery [105]. Rectal examination is also reportedly painful, and most patients show fever of 38 °C to 38.5 °C, rarely accompanied by chills. In chronic cases, mild fever may persist for several weeks. Anorexia, vomiting, and modified bowel transit (diarrhea or constipation) are also present in about half the patients. An important finding is the palpation of a tumor-like mass in the right lower quadrant of the abdomen that can be confused with a malignancy. There are several reports of patients undergoing biopsy or surgery with a diagnosis of cancer, with a later confirmation of inflammatory disease. It is noteworthy that parasitic structures in histological sections are not easily identified amid inflammatory tumor-like lesions [105–107].

Clinical signs in extraintestinal anatomical locations might appear during AA. Some patients complain of pain in the right upper quadrant. In these cases, the liver is usually enlarged and tender to palpation [108]. During laparoscopy, small yellowish spots or granulomas are observed on the surface of the liver. Most patients have hepatic isolated or combined involvement with intestinal angiostrongyliasis [58, 108]. In addition, erratic migration of larvae may result in adult development in other arterial territories. When the testicle is involved, the patient experiences acute pain, accompanied by redness and then purple discoloration. Eosinophilia and leukocytosis are also conspicuous. All patients with testicular necrosis have been misdiagnosed as having testicular torsion, and the correct diagnosis is assessed only after surgery [109].

Angiostrongylid intravascular parasites may be involved in complex host- or worm-modulated coagulation mechanisms, which raises an alert for more detailed studies on pathogenesis and coagulation. With this hypothetical role for coagulation imbalance in AA pathogenesis, Rodriguez and collaborators have explored the use of enoxaparin to avoid thrombotic complications, a very interesting subject for investigation [110].

Rare clinical presentations are worth mentioning due to the severity that they imply and to increase awareness of potential complications of this disease and its differential diagnosis. Distal lower limb arterial thrombosis leading to ischemic necrosis and amputation may occur (Morera, personal communication, October 1997). Also, severe gastrointestinal bleeding may result from the intestinal lesions [26]. Moreover, a suspected case of *A. costaricensis* infection was reported with severe pulmonary embolism, even though the parasites could not be detected [111]. *Angiostrongylus costaricensis* worms are not expected to occur in pulmonary arteries, which are the usual location of *A. cantonensis* in rodents, but not in humans. Therefore, in this case, it is suspected that the patient might have been infected with *A. cantonensis* rather than *A. costaricensis.* Intra-arterial nematode sections have been rarely registered in ectopic locations, such as (i) inside the mesenteric arteries in a patient living in an endemic area for *A. cantonensis* [112] and (ii) *A. costaricensis* inside pulmonary arteries in a patient living in Guatemala (Argueta-Sandoval, personal communication, July 2018). Guatemala, like most coastal areas in the Americas, may have had an introduction of *A. cantonensis* and thus may have the circulation of both parasites. Interestingly, the first demonstration of *A. cantonensis* active transmission in the Americas and Caribbean region was a collaboration between Pedro Morera and Cuban colleagues after an investigation of an eosinophilic meningitis outbreak in Cuba [113]. Therefore, clinical manifestations resembling AA without getting to a confirmatory diagnosis must be carefully studied to rule out the possibility of *A. cantonensis* infection in patients.

### Pathological findings

**Fig. 5:**
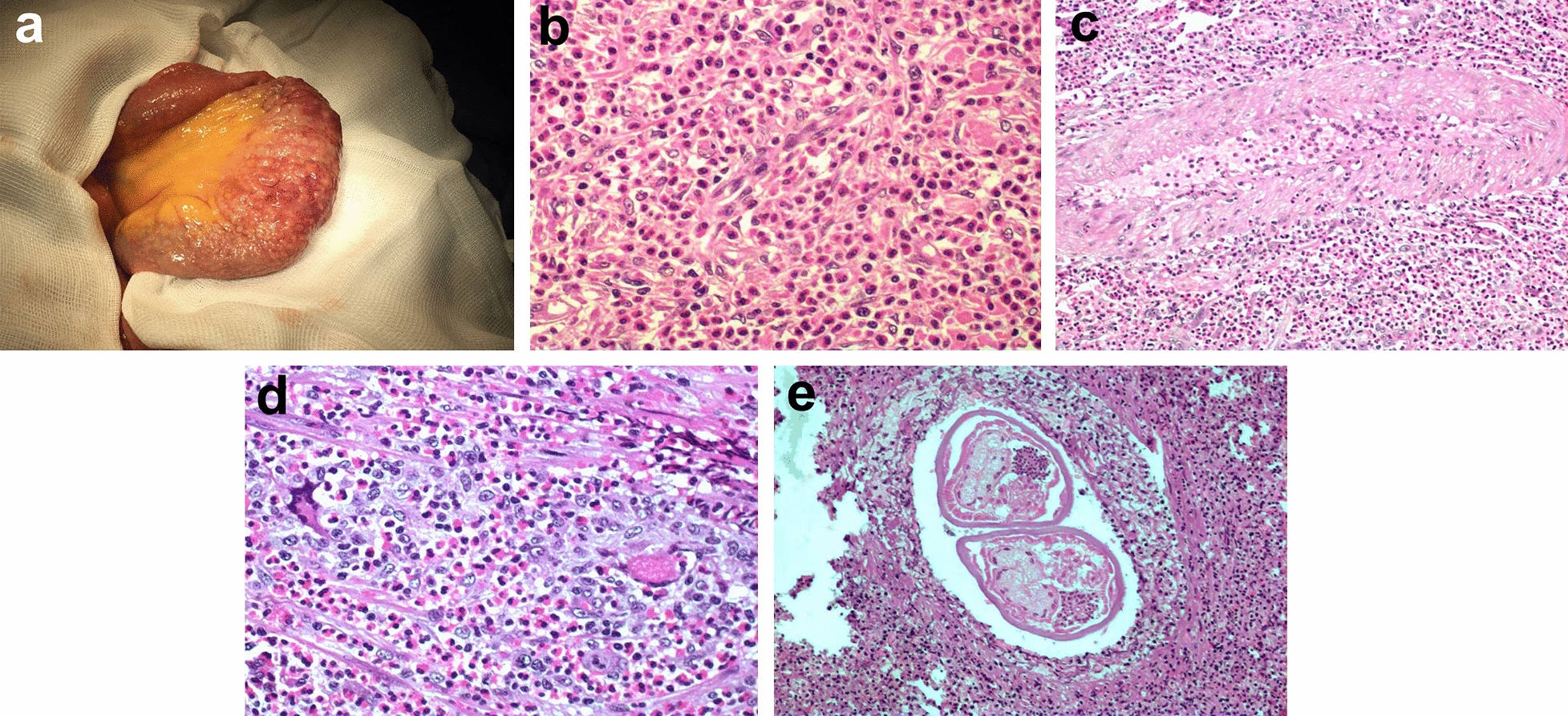
Histopathology findings during *Angiostrongylus costaricensis* infection in humans. **a** Segmental lesion in the small bowel with granulation in the serosal surface. **b** Intense and diffuse eosinophilic infiltration in gut mucosa (magnification: ×400). **c** Eosinophilic arteritis (magnification: ×100). **d** Granulomatous reaction engulfing egg in capillary lumen (magnification: ×400). **e** Adult worms inside arterial lumen (magnification: ×100)

Abdominal angiostrongyliasis is characterized by a strong inflammatory response leading to macroscopic and microscopic alterations of the affected tissues. Reported macroscopic findings include ischemic intestinal infarction, segmental thickening of the small bowel mimicking Crohn's disease (Fig. 5a), nodule-like pseudotumors located in the colon, and acute appendicitis [114]. Moreover, microscopic tissue alterations include intense and diffuse eosinophilic infiltration (Fig. 5b), eosinophilic vasculitis, especially eosinophilic arteritis (Fig. 5c), and strong granulomatous reaction, generally associated with retention of eggs or larvae in the capillary lumen (Fig. 5d) [110]. In addition, adult specimens are found in the arterial lumen of the affected organ (Fig. 5d), which can contain intact or partially degenerated worms. The vascular structure can be undisturbed or elicit alterations such as eosinophilic arteritis, granulomas, necrosis, and thrombi. Thrombotic events, vasculitis, and vascular granulomas culminate in ischemia and infarction, which can all lead to intestinal wall perforation [110].

### Immunopathology

AA is characterized by a strong inflammatory response involving mainly the ileocecal region [115]. Other anatomical regions reported to be affected by *A. costaricensis*-mediated inflammation include the liver, testicles, omentum, abdominal wall hernia, and colon [33, 69, 116]. The presence of eggs and adult worms triggers a strong type 2 inflammatory response characterized by the formation of eosinophilic granulomas and vasculitis leading to tissue damage.

The impact of the immunological alterations in the pathology of AA becomes evident in the clinical scenario, as well as in the increased eosinophilia and local alterations observed, such as chronic inflammation and eosinophilic granulomatous responses [33]. Non-natural hosts of *A. costaricensis*, permissive to infection with L3 larvae, develop a marked IgE-mast cell activation and massive tissue eosinophil infiltration [117]. Therefore, in non-permissive hosts such as humans, a stronger immunopathological response is expected.

Even though the immunopathological mechanisms in humans are yet to be elucidated, it is believed that egg release may be a crucial event triggering an inflammatory response that leads to the granuloma formation and tissue damage. Inflammatory and eosinophilic granulomatous responses are present in the anatomical areas containing eggs of *A. costaricensis* [33]. Due to the strong immune response against egg molecules, soluble egg antigens have been proposed as candidates for the specific diagnosis of AA [118]. In contrast, adult worm extracts show a different immunological shift, as a strong regulatory response was found to be elicited towards blocking allergic pulmonary responses in mice [119–121]. These findings suggest that distinct responses are induced during *A. costaricensis* infections due to their development from larvae to sexually mature nematodes releasing eggs to the host tissues with the concomitant antigen release from each of these stages.

Unraveling the mechanisms underlying the immune regulation generated by *A. costaricensis* is a challenging task considering the parasite’s life cycle inside the definitive host and host specificity. Most studies focused on the immune regulation by *A. costaricensis* have been performed on several mouse models, which have resulted in different susceptibility and immune responses to this parasite [52]. The mouse H2 haplotype determines the strain susceptibility to the parasite. For instance, H2b haplotype strains (C57BL/6, C57BL/10) are associated with high survival rates and resistance, whereas H2d haplotype strains (BALB/c, DBA/2) are linked to low survival rates and susceptibility [122]. Furthermore, studies using experimentally infected mice ex vivo spleen cell proliferation assays have shown differences in the immune responses elicited to the parasite by the different mouse strains. After stimulation with *A. costaricensis* adult worm antigen, the response was higher in C57BL/6 mice than in BALB/c mice, suggesting an association between higher susceptibility and cellular hyporesponsiveness [123]. However, other studies showed that BALB/c mice developed well-formed granulomas in the intestine [124], and that the survival rate of C57BL/6 mice was not affected by major histocompatibility complex (MHC)-II deficiency and its associated cellular and humoral unresponsiveness [125]. This indicates that further research is necessary to elucidate the immune mechanisms related to the different degrees of susceptibility of specific mouse strains. Even though mouse models are useful for studying the immunopathology of *A. costaricensis* infection, distinct strain responses and differences from the infection in humans represent important limitations to understanding the immunological features leading to the pathological alterations observed in AA.

### Diagnosis

Clinical diagnosis is based on the characteristic features of abdominal pain, fever, and peripheral eosinophilia [4]. There are three main syndromic arrangements of clinical manifestations: (i) acute abdominal pain associated with intestinal perforation, (ii) subacute disease with tumor detection in the ileocecal transition, and (iii) more subtle chronic inflammatory disease [106]. The former two conditions are associated with ischemic-congestive and pseudo-neoplastic patterns [114]. Notably, these symptoms and findings can be confused with those of appendicitis. Interestingly, the appendix is frequently involved in the infection, but the diagnosis of AA is usually made at surgery. Imaging-based examination can demonstrate suggestive findings for AA, usually localized in the terminal ileum, cecum, appendix, and ascending colon. The intestinal wall may show spasticity and thickening with variable degrees of lumen reduction producing a tumor resembling cancer [33]. Eggs and larvae do not usually appear in the stools; therefore, a fecal examination has no real diagnostic value.

Histopathological analysis is considered the current gold standard method for diagnosing AA. The confirmation of the diagnosis depends on the observation of eggs or larvae within the tissues, or *A. costaricensis* adult worms located in the arterial lumen or its branches [114, 126]. The diagnosis is presumptive when no parasitic structures are observed but there are histological changes that can guide the final diagnosis, such as eosinophilic vasculitis, eosinophilic arteritis, prominent eosinophilic infiltrate, and peri- or intravascular granulomas. An appropriate microscopic analysis to confirm the diagnosis requires a collection of multiple samples from different affected anatomical areas, even the entire lesion or the surgically removed tissue in FFPE blocks. Since this strategy is time-consuming and requires an experienced pathologist, it is important to consider different approaches to simplify the diagnosis. A conventional PCR targeting fragments of a 66-kDa protein of *A. cantonensis* in FFPE samples has been developed to confirm the identity of parasitic structures and unclassified samples with presumptive diagnosis featuring granulomatous reactions and clinical signs of AA [98].

Although a few patients may have no hematological abnormalities, leukocytosis and eosinophilia are usually present. White blood cell counts usually range between 15,000 and 50,000/mm^3^ and eosinophilia from 20 to 50%. Leukocytosis has been as high as 169,000/mm^3^, with 91% eosinophilia [106, 107].

While confirmation of AA is only achieved by histopathology and molecular assays, serological techniques with detection of antibodies have been the main stem in the diagnostic pipeline of AA. Currently, a latex agglutination test is performed in the Parasitology Reference National Center of Parasitology in the INCIENSA (Health and Nutrition Research and Teaching Institute of Costa Rica) in Costa Rica [37]. One main limitation of serological tests is the lack of well-characterized true-positive serum samples of patients with AA, due to the difficulty in obtaining sera from patients with histopathological demonstration of worms and/or eggs. Homologous or heterologous (i.e. from *A. cantonensis*) female worm and egg antigens have been employed in immunoenzymatic assays, with estimates of sensitivity ranging from 88 to 91% and specificity from 76 to 88% [118, 127, 128], and some of these studies reported cross-reaction when using sera from *Strongyloides stercoralis*-infected patients. IgG ELISA with heterologous crude female antigen has been used for the last decade at the parasitology reference laboratory located at the Pontifícia Universidade Católica do Rio Grande do Sul in Brazil, with estimated sensitivity of 88.4% and specificity of 78.7% [127]. It has also been demonstrated that IgG ELISA reactivity gradually decreases and disappears after 12 months of follow-up [129]. These relatively short dynamics of humoral response in AA may aid the interpretation of serological reactivity, especially for treatment control and epidemiological surveys.

The production of homologous recombinant proteins has been useful for the design of novel serological tools. A recombinant galectin from *A. cantonensis* has been used as an antigen for the diagnosis of cerebral angiostrongyliasis in a point-of-care immunochromatographic test. Using this assay, anti-*A. costaricensis* antibodies were identified in 11 out of 12 histology-proven infections and stands as a promising serological tool for AA [130]. Other homologous recombinant antigens have been under study, and a combination of several antigens [131, 132] and strategies to improve specificity [132] would be the way forward for antibody detection in AA. Nucleic acid detection of a 66-kDa protein in both serum and tissues has been investigated, but extended evaluation of performance is needed before their role in diagnosis is firmly established [95, 98].

### Treatment

Surgical intervention remains the most effective strategy for treating acute AA, since no convincing data have been obtained from the use of anthelmintic drugs [133]. However, as knowledge of this infection increases, the number of cases resolved without surgery is also increasing. There is no clear efficacy demonstration of anti-parasitic, anti-inflammatory, or anti-thrombotic drugs in angiostrongylid helminthic human infections, even for the treatment of cerebral angiostrongyliasis caused by *A. cantonensis*, where a greater number of studies have been done [134, 135]. In vitro and in vivo trials in experimentally *A. costaricensis-*infected rats demonstrate that parasites are stimulated by thiabendazole, levamisole, and diethylcarbamazine, rather than killed, causing erratic migrations and worsening of the lesions [133]. Although this finding was not reproduced with mebendazole [135], chemotherapy is not recommended during *A. costaricensis* infections, while corticosteroids have a clear role in treatment only for *A. cantonensis* infections [134].

### Control measures and prophylaxis

Controlling natural definitive and intermediate host populations of *A. costaricensis*, through cooperative work with small and large farmers, is a critical measure for reducing the risk of disease. Mollusk populations can be regulated with chemical or biological control. The former should be avoided, since other animal species can be affected. Therefore, biological control should be meticulously designed to obtain a selective and effective reduction in host populations [136]. Additionally, controlling the introduction of mollusk species in geographical regions where they can become pests and potential intermediate hosts of this nematode is strongly recommended.

Transmission to humans is achieved with the consumption of fruits and vegetables contaminated with the intermediate hosts or, less likely, with the secretions derived from them [16]. Studies have demonstrated that *A. costaricensis* L3 larvae remain viable at 5 °C for less than 14 days, and some become inactive from day 7 of incubation. Therefore, refrigeration of potentially contaminated vegetables should not be used as the sole measure for preventing infection with this parasite [137]. Instead, disinfection with 1.5% chloride solution for one hour is recommended to attain larvicidal effects [138]. In addition, certain cultural traditions feature the eating of raw snails or preparing meals based on them [40], which can trigger infection with *A. costaricensis*. Therefore, providing education to the general population regarding the correct disinfection of fruits and vegetables before consumption and increasing the awareness of potential transmittal of parasites by raw meals is extremely important.

## Conclusions

Over the last 50 years, a great number of studies have elucidated several aspects of *A. costaricensis* ecology, taxonomy, pathology, and molecular biology, and have provided novel ways to diagnose AA. Nevertheless, knowledge gaps in the taxonomic positioning of *A. costaricensis*, especially for those specimens collected from different hosts and geographical locations, and the potential circulation of novel *Angiostrongylus* spp. are still unfilled. Also, the role of introduced and invasive mollusks as intermediate hosts of this parasite poses a threat for spreading and increasing *A. costaricensis* infection to humans.

The most efficient way to treat AA in humans is still surgical intervention, since anthelmintics have not proven useful for eliminating *A. costaricensis* infection. Therefore, it is necessary to analyze the immunological response during AA, since this will help us understand the intricate mechanisms underlying the infection and hopefully will render novel ways to treat and diagnose AA. Additionally, with a deep learning of *A. costaricensis -*omics, we will be able to elucidate the metabolic pathways, host–parasite interactions, and uniquely expressed protein for the design of sensitive and specific diagnostic tools. This profound knowledge will be gained only with collaboration among physicians, microbiologists, veterinarians, biologists, and ecologists from all the Americas.

## Data Availability

Not applicable.

## Abbreviations

AA: Abdominal angiostrongyliasis
BALB/c: Albino laboratory-bred mouse strain
C57BL/6: Inbred mouse strain known as C57 black 6
*cox*1: Cytochrome oxidase subunit 1
ELISA: Enzyme-linked immunoassay
FFPE: Formalin-fixed paraffin-embedded tissues
INCIENSA: Health and Nutrition Research and Teaching Institute of Costa Rica
IFN-γ: Interferon gamma
IL-17: Interleukin 17
ITS-2: Internal transcribed spacer 2
L1: First larval stage
L2: Second larval stage
L3: Third larval stage
MS: Mass spectrometry
PCG: Protein-coding gene
PCR: Polymerase chain reaction
rDNA: Ribosomal DNA
PP2A: Serine/threonine phosphatase 2 A
RFLP: Restriction fragment length polymorphism
